# The association between crystallopathy and postoperative complications following total hip and knee arthroplasty: a systematic review and meta-analysis

**DOI:** 10.1097/MS9.0000000000004555

**Published:** 2025-12-18

**Authors:** Yashar Mashayekhi, Amir-Mohammad Asgari, Mohammad-Taha Pahlevan-Fallahy, Ronak Jalali, Sara Baba-Aissa, Farhad Shaker

**Affiliations:** a Department of Orthopaedic, Leicester University Hospital, Leicester, UK; b School of Medicine, Kermanshah University of Medical Sciences, Kermanshah, Iran; c School of Medicine, Tehran University of Medical Sciences, Tehran, Iran; d School of Medicine, Golestan Beheshti University of Medical Sciences, Gorgan, Iran; e Internal Medicine Department, Leicester University Hospital, Leicester, UK

**Keywords:** arthroplasty, crystallopathy, THA, TJA, TKA

## Abstract

**Background:**

Crystallopathies, including gout and calcium pyrophosphate deposition (CPPD), are prevalent conditions that complicate total joint arthroplasty (TJA). This study aims to evaluate the impact of crystallopathy on postoperative outcomes following total hip and knee arthroplasty, including complication rates, revision risks, and functional recovery.

**Methods:**

A systematic review and meta-analysis were conducted in accordance with PRISMA 2020 guidelines. Studies comparing the outcomes in adult patients undergoing total hip or knee arthroplasty between cases with and without crystallopathy were included. A random-effects meta-analysis was performed using R software version 4.4.2. Sensitivity and subgroup analyses were conducted to identify outliers and assess effect sizes based on crystallopathy type and arthroplasty type.

**Results:**

A total of 23 267 319 TJA cases, including 921 787 crystallopathy group cases, were analyzed. The meta-analysis revealed a significantly higher rate of wound complications (OR = 1.41, 95% CI: 1.03–1.91), prosthetic joint infection (PJI) (OR = 1.97, 95% CI: 1.01–3.83), thromboembolic events (OR = 1.19, 95% CI: 1.06–1.33), and non-home discharge rates (OR = 1.20, 95% CI: 1.02–1.40) in the crystallopathy group, all of which lost significance in sensitivity analysis. However, no significant differences were observed in in-hospital mortality, transfusion needs, myocardial infarction (MI), revision surgery, length of stay (LOS), hospital charges, and emergency department (ED) visits.

**Conclusion:**

Patients with crystallopathies might be at an increased risk of wound complications, PJI, thromboembolic events, and non-home discharge. These findings emphasize the importance of preoperative risk assessment and personalized perioperative management for this patient group.

## Introduction

Crystallopathies, such as gout, calcium pyrophosphate dihydrate deposition (CPPD), and basic calcium phosphate deposition (BCPD), result from microcrystal deposition in joints and tissues, causing inflammation, tissue remodeling, and mechanical obstruction^[1]^. These conditions are highly prevalent, with gout affecting 0.68% to 14% of adults globally^[2]^ and CPPD prevalence reaching up to 13%, particularly in individuals over 70 years^[3]^. BCPD is increasingly recognized in joint pathology. Crystallopathies frequently coexist with osteoarthritis, complicating total joint arthroplasty (TJA), a common procedure for severe joint disease^[4]^. The rising incidence of TJA and metabolic disorders like hyperuricemia underscores the growing relevance of crystallopathies in surgical outcomes^[5]^.

**HIGHLIGHTS**

- Crystal-induced arthropathies significantly increase the risk of postoperative complications in total joint arthroplasty (TJA), including wound complications, prosthetic joint infection (PJI), thromboembolic events, and non-home discharge.
- TJA cases revealed higher complication rates in patients with crystallopathies, but no significant difference in mortality, transfusion needs, or revision surgery.
- Subgroup analysis showed that gout patients had a higher risk of wound complications and PJI compared to those with calcium pyrophosphate deposition.
- Personalized perioperative management and preoperative risk assessment are crucial for patients with crystallopathies to mitigate postoperative risks and improve outcomes.

Recent studies highlight the diagnostic and therapeutic challenges of crystallopathies in arthroplasty. Crystal deposition can mimic prosthetic joint infection (PJI), presenting with pain, swelling, and erythema^[6]^. Approximately one-third of cases are initially misdiagnosed as PJI, leading to inappropriate antibiotic or surgical interventions^[7]^. Synovial fluid analysis is essential for accurate diagnosis, supported by imaging modalities like ultrasound or dual-energy CT^[8]^. While anti-inflammatory treatments are effective, the impact of crystallopathies on TJA outcomes, such as revisions or functional recovery, remains understudied^[9]^. While some of the previous studies suggest no significant differences in mortality, PJI, or wound complications between patients with and without calcium pyrophosphate deposition (CPPD)^[10–12]^; however, some other studies have reported a significantly higher rate of revision, PJI^[13–15]^. These inconsistencies highlight the need for further research with larger, standardized studies to clarify crystallopathies’ impact on TJA outcomes and improve diagnosis and treatment.

Given the diagnostic complexity and increasing prevalence of crystallopathies, a comprehensive evaluation of their effects on arthroplasty is warranted. Since some of these complications, like thromboembolism and PJI, might need additional interventions, there is a necessity for accurate statistics on whether the risk of these complications is increased or not. Thromboembolism, for example, might require the administration of certain anti-coagulant therapies, which might have drug interactions with the patients’ current anti-inflammatory regiment for crystallopathy. Also, PJI, one of the endpoints of the study, is one of the leading causes of revision surgery and is one of the important causes of patients’ dissatisfaction and if the risk proves to increase in this group of patients, it might need more aggressive surveillance and treatment strategies. This study also complies with the TITAN 2025 Guidelines on Transparency, Integrity, and the Responsible Use of Artificial Intelligence in Scholarly Publishing^[16]^. This meta-analysis aims to synthesize evidence on how crystallopathy influences TJA outcomes, including complication rates, revision risks, and functional scores, to inform clinical practice and identify research gaps.

## Materials and methods

This systematic review and meta-analysis adhered to the PRISMA 2020 guidelines^[17]^ and has been registered with PROSPERO under the code (CRD420251051578).

### Search strategy

A comprehensive search was conducted in the following electronic databases: PubMed, Embase, Web of Science, and Scopus, covering the period from inception to 30 March 2025. The search strategy employed the terms “crystallopathy,” “gout,” “pseudogout,” “calcium pyrophosphate,” “total joint arthroplasty,” “total hip arthroplasty,” and “total knee arthroplasty,” combined with relevant synonyms and Boolean operators (OR) and (AND) in the title and abstract fields. Reference lists of the included articles were manually searched for additional eligible studies.

The specific search strategies for each database are detailed in Supplemental Digital Content Table S1, available at: http://links.lww.com/MS9/B55.

### Eligibility criteria

Studies were eligible for inclusion if they adhered to the PECO framework and were not restricted by publication date or language. The population consisted of adult patients who underwent either total hip arthroplasty (THA) or total knee arthroplasty (TKA). The exposure group included patients diagnosed with crystallopathy (including gout and CPPD disease), as determined by clinical or laboratory criteria. The comparison group consisted of patients who underwent THA or TKA without crystallopathy.

The primary outcomes of interest were mortality and periprosthetic joint infection (PJI). Secondary Outcomes were hospital readmissions, cardiac complications, revision surgeries, length and costs of hospital stays, gastrointestinal complications, anemia, wound complications, non-home discharge, renal and urinary complications, thromboembolic events, and functional and motion-related outcomes.

Studies were excluded if they lacked sufficient data on the outcomes of interest, did not focus on THA or TKA, or did not compare patients with crystallopathy to a control group. Additionally, letters, book chapters, review articles, and qualitative studies were excluded.

### Study selection

The search results were imported into the Rayyan software for screening. Duplicates were removed, and two independent reviewers (A.M.A. and Y.M.) assessed the titles and abstracts of studies to identify those eligible for full-text review. Full-text articles were independently assessed by the same reviewers. Any discrepancies in the selection process were resolved by a third independent reviewer (F.Sh.).

### Data extraction

Data were extracted by two independent reviewers (R.J. and Y.M.) using a standardized form. The following information was extracted from each included study: author, year of publication, country, study design, sample size, patient demographics (age, gender), and outcomes of interest as outlined in the eligibility criteria.

### Quality assessment

The quality of the included studies was independently assessed by two reviewers (R.J. and M.T.P.F) using the Newcastle-Ottawa Scale (NOS)^[18]^ for cohort and case-control studies. The AXIS tool^[19]^ was utilized to assess the quality of cross-sectional studies. Any disagreements in quality assessment were resolved through discussion, or if necessary, by a third reviewer (A.M.A.).

### Statistical analysis

A random-effects meta-analysis was performed using the “meta” package in R software version 4.4.2. For dichotomous variables, outcomes were expressed as odds ratios (ORs), while continuous variables were reported as standardized mean differences (SMDs). The I^2^ statistic was used to assess heterogeneity, with I^2^ values greater than 30% indicating substantial heterogeneity. Sensitivity analyses were performed to investigate potential outliers; by excluding studies one by one and measuring whether the results would lose significance, the reliability of results can be measured along with the degree of heterogeneity. Subgroup analysis was conducted to investigate the effect size in each subgroup according to the type of crystallopathy (gout, CPPD, other) as well as the type of arthroplasty (TKA, THA) where each subgroup consisted of two or more studies. Publication bias was assessed using funnel plots and Harbord’s test. Statistical significance was defined as a *P*-value of <0.05.

## Results

### Study selection

**Figure 1.**
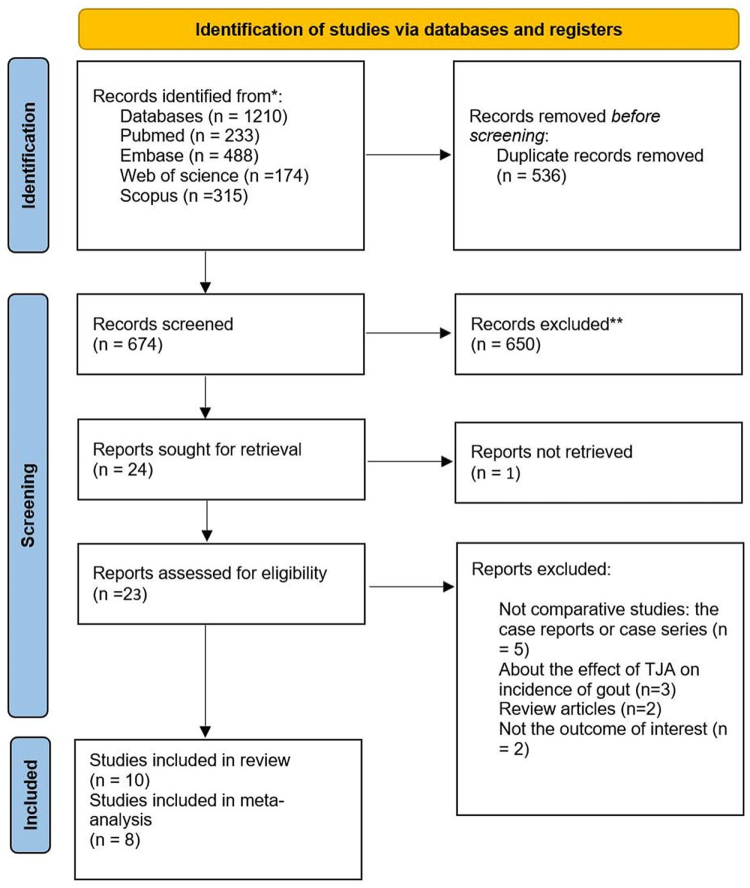
PRISMA flowchart.

After a systematic search of four electronic databases, including PubMed, Scopus, Web of Science, and Embase, and removal of duplicates, we identified 674 identical records. These records then underwent title/abstract screening, which resulted in detecting 23 to be relevant for full-text review. After subjecting a full-text review, 10 papers were^[10–15,20–23]^ considered eligible to be included in this systematic review, and eight^[10–15,20,22]^ were appropriate to be included in the meta-analysis (Fig. 1).

### Baseline characteristics and quality assessments

A total of 23 267 319 total joint arthroplasty cases (58.8% female) were included in our systematic review, of which 921 787 (3.8%) were crystallopathy cases, and 22 345 532 (96.0%) were control patients. The exposure group comprised gout (903 615; 98.0%) and CPPD (18 172; 1.9%) patients. Based on the available data, 63.8% of the study population underwent TKA, and 36.1% were THA cases.

The included studies were conducted in the USA^[10–13,15,20–23]^ and the Netherlands^[14]^. Furthermore, four studies used the NIS database^[10,11,15,22]^, two utilized the Medicare database^[20,23]^, one used the PearlDiver database^[13]^, and three were based on single-center datasets^[12,14,21]^. All of the included studies except Lee *et al*, Chen *et al*, and Williams *et al* used retrospective data from public databases which did not exactly define their diagnostic criteria; Lee *et al* defined CPPD diagnosis by noticing crystal deposits during surgery. Chen *et al* defined gout diagnosis as “if the patient had a diagnosis of gout by their internist prior to surgery or were receiving urate-lowering agents or colchicine.” Williams *et al* defined the diagnosis of CPPD by having visible chondrocalcinosis in knee X-rays. Regarding the study design, five were retrospective cohorts^[10,13,14,20,21]^, two were retrospective case-control^[12,23]^, and three were cross-sectional^[11,15,22]^. Two studies, those by Salt *et al* and Singh *et al* reported the use of fundings, while others did not report or reported no fundings. Table 1 comprehensively demonstrates an overview of the included studies’ baseline characteristics.

**Table 1:** Baseline characteristics of the included studies

Author date	Country	Study design	Dataset	Sample size	# of THA and TKA	# of Gout and CPPD	Age, mean (SD)	Males (%)	Follow-up
Willems (2019)	Netherlands	Retrospective Cohort	TKA patients at Spaarne Gasthuis, 2010–2011	408	0 & 408	0 & 63	68.4 (9.5)	143 (35.0%)	5 years
Parperis (2023)	USA	Cross Sectional	National Inpatient Sample (2006–2014)	4 111 808	4 111 808 & 0	217 926 & 6168	77 (NA)	1 472 027 (35.8%)	In-hospital
Rosas (2020)	USA	Case Control	Medicare data 2005–2014	15 238	0 & 15 238	7619 & 0	NA	6491 (42.6%)	90 days
Bradley (2021)	USA	Retrospective Cohort	Medicare database 2009–2013	1 135 749	0 & 1 135 749	64 738 & 0	NA	624 662 (55.0%)	1 year
Singh (2019)	USA	Cross Sectional	NIS 1998–2014	8 127 182	0 & 8 127 182	231 470 & 0	66.4 (NA)	2 982 676 (36.7%)	In-hospital
Lee (2014)	USA	Retrospective Cohort	Single surgeon series, 1992–2003	1500	0 & 1500	0 & 412	70 (NA)	511(35.4%)	57 months (mean)
Chen (2016)	USA	Case Control	Single institution database, 2000–2012	964	474 & 490	482 & 0	66.1 (10.6)	383 (79.5%)	90 days
Singh (2019)	USA	Retrospective Cohort	NIS 1998–2014	4 116 485	4 116 485 & 0	104 098 & 0	67 (NA)	1 776 722 (43.2%)	In-hospital
Parperis (2022)	USA	Cross Sectional	NIS 2006–2014	5 564 005	0 & 5 564 005	244 816 & 11 529	72 (NA)	2 570 570 (46.2%)	In-hospital
Zhang (2024)	USA	Retrospective Cohort	PearlDiver 2010–2022	193 980	193 980 & 0	32 466 & 0	NA	137 380 (70.8%)	2 years

CPPD, calcium pyrophosphate deposition disease; NIS, National Inpatient Sample; NA, not available; SD, standard deviation; THA, total hip arthroplasty; TKA, total knee arthroplasty

Seven studies were assessed based on NOS^[24]^, of which six^[11–14,20,23]^ showed a high methodological quality and one^[21]^ demonstrated a moderate methodological quality. Supplemental Digital Content Table S1, available at: http://links.lww.com/MS9/B55 and Supplemental Digital Content Table S2, available at: http://links.lww.com/MS9/B55 show all study’s NOS scores across selection, comparability, and outcomes domains. Three^[10,15,22]^ cross-sectional studies were assessed based on AXIS^[19]^, and all showed a high methodological quality (Supplemental Digital Content Tables S2–S4, available at: http://links.lww.com/MS9/B55).

### Meta-analyses

#### Primary outcomes

**Figure 2.**
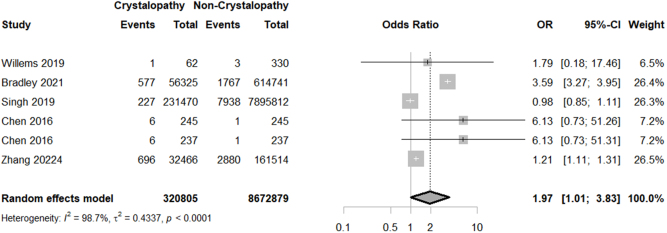
Forest plot of prosthetic joint infection rate comparison between crystallopathy group and control group undergoing total joint arthroplasty (OR with 95% CI).

Our meta-analysis demonstrated a higher rate of PJI [OR = 1.9708; 95% CI: (1.013–3.833); *P* = 0.0457; I^2^ = 98.7%] in the crystallopathy group (Fig. 2). However, performing a sensitivity analysis, by excluding Willems *et al*, Bradley *et al*, and both groups of the study by Chen *et al*, the difference of PJI rates between the groups lost significance (Supplemental Digital Content Figure S1, available at: http://links.lww.com/MS9/B55). Due to the high level of heterogeneity which did not resolve even after a sub-group analysis on the arthroplasty and crystallopathy type, interpretation of results must be done with caution (Fig. 2). However, performing a sensitivity analysis, by excluding Willems *et al*, Bradley *et al*, and Chen *et al*, demonstrated comparable odds of PJI between the control and crystallopathy group (Supplemental Digital Content Figure S1, available at: http://links.lww.com/MS9/B55).

**Figure 3.**
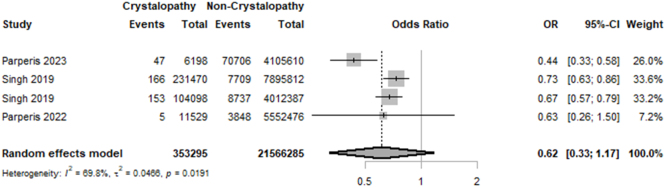
Forest plot of in-hospital mortality comparison between crystallopathy group and control group undergoing total joint arthroplasty (OR with 95% CI).

According to our analysis, there was no considerable difference in terms of in-hospital mortality (OR = 0.62; 95% CI: (0.33–1.17]; *P* = 0.08; I^2^ = 69.8%) between the two groups (Fig. 3). These results remained consistent after excluding each of the studies one by one (Supplemental Digital Content Figure S2, available at: http://links.lww.com/MS9/B55).

#### Secondary outcomes

Out of the intended secondary outcomes, no study reported on gastrointestinal and anemia. Renal and urinary complications were reported in only two studies; Chen *et al* reported significantly increased rates of renal complications in both TKA and THA patients and no significant difference between two groups in both TKA and THA patients in terms of urinary complications^[12]^. In addition, Rosas *et al* showed no significant difference between gout and non-gout patients undergoing TKA in terms of UTI incidence rate^[23]^. Readmission rate was only investigated by Chen *et al*, who showed a significantly higher rate of 90-day readmission in the gout group among patients undergoing THA, while no significant difference was observed between the gout and non-gout groups in patients undergoing TKA^[12]^. Other secondary medical, surgical, and hospitalization-related outcomes were reported and included in the meta-analysis.

##### Surgical and medical complications

**Figure 4.**
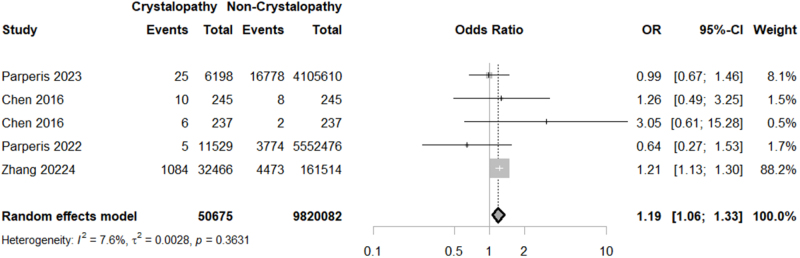
Forest plot of thromboembolism rate comparison between crystallopathy group and control group undergoing total joint arthroplasty (OR with 95% CI).

**Figure 5.**
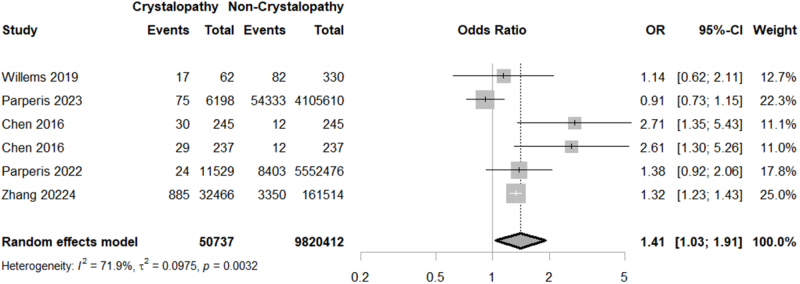
Forest plot of wound complication rate comparison between crystallopathy group and control group undergoing total joint arthroplasty (OR with 95% CI).

Considering surgical and medical complications, our study unveiled a significantly higher rate of thromboembolism (OR = 1.1859; 95% CI: 1.0565–1.3312; *P* = 0.0038; I^2^ = 7.6%) and wound complications (OR = 1.4054; 95% CI: 1.0323–1.9132; *P* = 0.0306; I^2^ = 71.9%) in crystallopathy group (Figs 4 and 5). However, there was no significant difference between the two groups regarding transfusion (OR = 1.1125; 95% CI: 0.9040–1.3691; *P* = 0.3139; I^2^ = 96.8%), myocardial infarction (MI) (OR = 1.0955; 95% CI: 0.8014–1.4974; *P* = 0.5674; I^2^ = 97.4%), and revision surgery (OR = 1.48; 95% CI: 0.84–2.62; *P* = 0.17; I^2^ = 99.3%) (Supplemental Digital Content Figures S3–S5, available at: http://links.lww.com/MS9/B55).

Furthermore, Rosas *et al*^[23]^ reported that compared to the control group, infection, cardiac arrest, pneumonia, hematoma, and capsulitis incidence were significantly higher in gout cases undergoing TKA, while pulmonary embolism was considerably lower in this group of patients.

Performing sensitivity analyses, it was shown that thromboembolism was no more significant after excluding the Chen *et al* and Zhang *et al* studies^[12,13]^. In addition, wound complications turned out to be comparable between the two groups following the exclusion of Chen *et al*, Parperis *et al*, and Zhang *et al*^[12,13,22]^ studies. The results of sensitivity analyses of wound complications, thromboembolism, transfusion, MI, and revision surgery are added in the supplementary material (Supplemental Digital Content Figures S6–S10, available at: http://links.lww.com/MS9/B55).

##### Hospitalization-related outcomes

**Figure 6.**
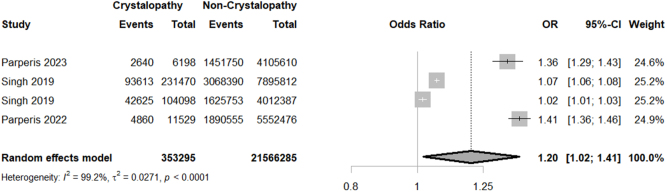
Forest plot of non-home discharge rate comparison between crystallopathy group and control group undergoing total joint arthroplasty (OR with 95% CI).

Regarding hospitalization-related outcomes, while our study showed a higher rate of non-home discharge (OR = 1.1998; 95% CI: 1.0203–1.4109; *P* = 0.0276; I^2^ = 99.2%) (Fig. 6) in crystallopathy group, there was no considerable difference between the two groups of patients concerning emergency department (ED) Visit (OR = 0.9631; 95% CI: 0.6596–1.4062; *P* = 0.8457; I^2^ = 38.6%), hospital charges (SMD = 0.0061; 95% CI: −0.0086–0.0209; *P* = 0.4139; I^2^ = 0.0%), length of stay (SMD = 0.0317; 95% CI: −0.0045–0.0679; *P* = 0.0859; I^2^ = 92.6%) (Supplemental Digital Content Figures S11–S13, available at: http://links.lww.com/MS9/B55).

Concerning sensitivity analysis, it was shown that NHD was no more significantly higher in the crystallopathy group after the exclusion of the Parperis *et al* study. However, the result of ED and hospital charges remained consistent after performing leave-one-out analysis. LOS remained comparable between the two groups after exclusion of all studies except for the Singh *et al* study, which resulted in significantly higher LOS in the crystallopathy group (Supplemental Digital Content Figures S14–S17, available at: http://links.lww.com/MS9/B55).

**Figure 7.**
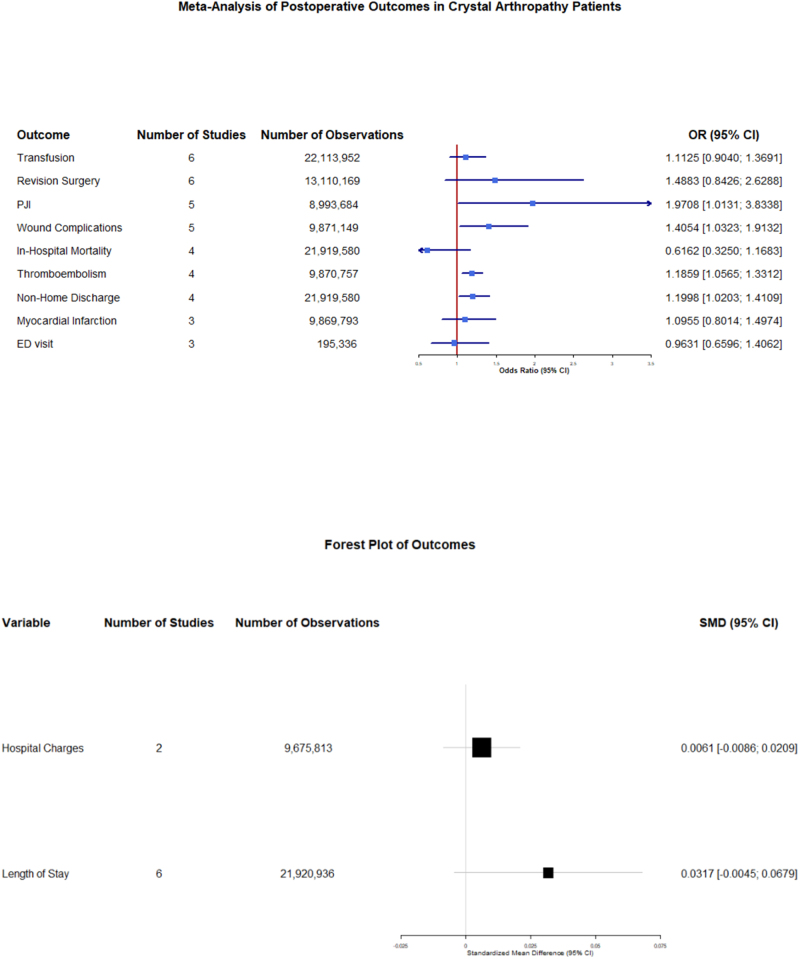
Summary of all outcomes meta-analysis.

Figure 7 summarizes the results of our meta-analysis and illustrates the crystallopathy-associated risk for each outcome and the investigated population size.

##### Functional and motion-related outcomes

Lee et al^[21]^ reported no significant difference in terms of Knee Society scores (KSS) and range of motion (ROM) between patients with and without chondrocalcinosis undergoing TKA. However, a decrease in ROM and KSS compared to the control group was observed in a subpopulation of cases who had a history of undergoing synovectomy for proliferative synovitis.

Furthermore, Willems *et al* ^[14]^ reported that there was no considerable difference in terms of Oxford Knee Score (OKS), KSS, and Algo functional Index, between cases with and without chondrocalcinosis who underwent TKA.

#### Subgroup analysis

##### Subgroup analysis based on the type of arthroplasty

There were no significant between-group differences for any of the outcomes (Supplemental Digital Content Figures S18–S28, available at: http://links.lww.com/MS9/B55). Table 2 comprehensively illustrates the subgroup differences regarding the type of arthroplasty for each of the outcomes.

**Table 2:** Subgroup analysis results based on type of arthroplasty

Outcome	Subgroup	OR/SMD	95% CI	I^2^	Between-group *P*-value
PJI	TKA	OR = 2.1590	[0.8693–5.3621]	98.8%	0.8802
	THA	OR = 1.8956	[0.4550–7.8974]	55.4%	
In-hospital mortality	TKA	OR = 0.7308	[0.00–inf]	0.0%	0.5633
	THA	OR = 0.5516	[0.0195–15.6183]	85.2%	
Transfusion	TKA	OR = 1.1202	[0.7442–1.6862]	0.0%	0.9858
	THA	OR = 1.1257	[0.7945–1.5950]	97.7%	
Revision surgery	TKA	OR = 1.1202	[0.7442–1.6862]	0.0%	0.9858
	THA	OR = 1.1257	[0.7945–1.5950]	97.7%	
Thromboembolism	TKA	OR = 0.8752	[0.4497–1.7033]	6.5%	0.3459
	THA	OR = 1.2075	[1.1298–1.2906]	13.0%	
Wound complications	TKA	OR = 1.5448	[1.0069–2.3700]	46.2%	0.6875
	THA	OR = 1.3474	[0.8087–2.2448]	84.8%	
Myocardial infarction	TKA	OR = 1.4768	[1.3626–1.6005]	–	–
	THA	OR = 0.9265	[0.8021–1.0703]	61.1%	
ED visit	TKA	OR = 0.7111	[0.3800–1.3304]	0.0%	–
	THA	OR = 1.1101	[0.7669–1.6070]	37.5%	
Non-home discharge	TKA	OR = 1.2273	[0.9341–1.6125]	99.5%	0.8236
	THA	OR = 1.1738	[0.8859–1.5551]	99.1%	
Hospital charges	TKA	SMD = 0.0021	[−0.0161–0.0204]	–	–
	THA	SMD = 0.0136	[−0.0113–0.0385]	–	
Length of stay	TKA	SMD = 0.0308	[−0.0153–0.0769]	90.7%	0.9524
	THA	SMD = 0.0333	[−0.0352–0.1018]	95.3%	

CI, confidence interval; ED, emergency department; I2, heterogeneity index; inf, infinity; OR, odds ratio; PJI, periprosthetic joint infection; SMD, standardized mean difference; THA, total hip arthroplasty; TKA, total knee arthroplasty

##### Subgroup analysis based on the type of crystallopathy

Subgroup analysis based on the type of crystallopathy unveiled a significantly higher risk of NHD (OR of 1.39 vs. 1.04; P-value < 0.0001) and a significantly more extended LOS (SMD of 0.06 vs. 0.00; P-value < 0.0001) in the CPPD subgroup compared to gout. No significant between-group differences for any of the other outcomes were observed (Supplemental Digital Content Figure S29-S38, available at: http://links.lww.com/MS9/B55). Table 3 demonstrates the subgroup analysis results regarding the type of crystallopathy for each of the outcomes.

**Table 3:** Subgroup analysis results based on type of crystallopathy

Outcome	Subgroup	OR/SMD	95% CI	I^2^	Between-group *P*-value
PJI	CPPD	OR = 1.7869	[0.1828–17.4632]	–	0.9214
	Gout	OR = 2.0157	[0.9743–4.1700]	98.9%	
In-hospital mortality	CPPD	OR = 0.4515	[0.00–inf]	0.0%	0.2755
	Gout	OR = 0.7050	[0.3319–1.4974]	0.0%	
Transfusion	CPPD	OR = 1.7464	[0.7215–4.2276]	0.0%	0.3071
	Gout	OR = 1.0874	[0.8788–1.3455]	98.7%	
Revision surgery	CPPD	OR = 1.7464	[0.7215-4.2276]	0.0%	0.3071
	Gout	OR = 1.0874	[0.8788–1.3455]	98.7%	
Thromboembolism	CPPD	OR = 0.9175	[0.6409–1.3134]	0.0%	0.1316
	Gout	OR = 1.2149	[1.1359–1.2994]	0.0%	
Wound complications	CPPD	OR = 1.0776	[0.8073–1.4385]	37.1%	0.0624
	Gout	OR = 1.9058	[1.1269–3.2231]	73.6%	
Myocardial infarction	CPPD	OR = 1.1356	[0.6781–1.9019]	98.6%	0.6809
	Gout	OR = 1.0140	[0.8642–1.1898]	–	
ED visit	CPPD	OR = 0.4904	[0.1451–1.6571]	–	0.2308
	Gout	OR = 1.0605	[0.7628–1.4744]	27.5%	
Non-home discharge	CPPD	OR = 1.3890	[1.3367–1.4433]	36.0%	< 0.0001
	Gout	OR = 1.0431	[0.9948–1.0937]	97.5%	
Length of stay	CPPD	SMD = 0.0635	[0.0440–0.0829]	0.0%	< 0.0001
	Gout	SMD = − 0.0030	[−0.0139–0.0079]	70.5%	

CI, confidence interval; CPPD, calcium pyrophosphate deposition disease; ED, emergency department; I2, heterogeneity index; inf, infinity; OR, odds ratio; PJI, periprosthetic joint infection; SMD, standardized mean difference.

#### Publication bias

Publication bias, investigated by linear regression test of funnel plot asymmetry, was insignificant for all of the outcomes except for ED, which showed a significant funnel asymmetry (*P*-value of 0.009). Table 4 reports the P-value of funnel asymmetry for all outcomes.

**Table 4:** *P*-value of Harbord’s test of funnel plot asymmetry for all outcomes

Outcome	Funnel plot asymmetry test *P*-value
Prosthetic joint infection	0.7014
In-hospital mortality	0.6550
Transfusion	0.6952
Revision surgery	0.6952
Thromboembolism	0.6510
Wound complications	0.7283
Myocardial infarction (MI)	0.9682
Emergency department (ED) visit	**0.0098**
Non-Home Discharge	0.1956
Hospital charges	—
Length of stay	0.2219

Bold p-values indicate statistical significance (p < 0.05).

Discussion

The findings of our study suggest that patients with crystallopathies have significantly increased odds of wound complications, PJI, thromboembolism, and non-home discharge when compared to those without; these outcomes, however, lost significance in sensitivity analysis when certain studies were removed. Differences in other outcomes, such as myocardial infarction, transfusion need, hospital charges, and mortality, were not significant. Considering the high number of patients with crystallopathies in our study (921 787) and in the general population, the difference in the risk of complications can be huge; the absolute risk difference for PJI, wound complications, NHD, and thromboembolism is 0.0299, 0.0213, 0.0431, and 0.0017, respectively.

Our analysis revealed a significantly higher risk of both wound complications and PJI in patients with crystallopathies, with a pooled OR of 1.41 (95% CI: 1.03–1.91; *P* = 0.0306) for wound complications, and an OR of 1.97 (95% CI: 1.01–3.83; *P* = 0.0457). Subgroup analysis showed that patients with gout had a non-significantly higher risk, compared to those with CPPD for both (*P* = 0.06, 0.12, respectively, for wound complications and PJI). Although the non-significance is marginal, this might suggest that the mechanism by which crystallopathies might affect wound healing and local immune response might be mediated by the inflammation, rather than the pathogenic mechanism by which crystal deposition occurred. Monosodium urate crystals in gout and calcium pyrophosphate crystals in CPPD are both known to cause a sterile inflammatory response, causing the activation of NLRP3 inflammasome, leading to the release of pro-inflammatory cytokines like IL-1, TNF-α, and IL-6^[25]^. From there, an increased inflammatory response might lead to the disruption in different stages of wound healing, namely fibroblast function and angiogenesis. This persistent inflammatory condition can impair tissue regeneration, delay epithelialization, and reduce local vascularization, thereby predisposing surgical wounds to infection. Moreover, the resulted immune dysregulation may compromise bacterial clearance at the surgery site, further increasing susceptibility to PJI. Prior intra-articular damage from flares might also create a more vulnerable intra-articular space post-op. Although the anatomic differences between the knee and hip joint might affect the healing process and infection risk, the differences between THA and TKA subgroups in our study were not significant (*P* = 0.68, 0.88, respectively, for wound complications and PJI). It also should be emphasized that crystal flares can closely mimic PJI in both clinical presentation and laboratory or imaging findings, having a risk of diagnostic misclassification in studies. A recent systematic review of crystal-induced arthritis in prosthetic joints claimed that about one-third of cases were initially considered PJI, and 35% even underwent surgical intervention or antibiotic therapy before crystals were identified^[26]^. Many PJI definitions (for example, MSIS, EBJIS) rely on overlapping criteria such as high CRP or ESR, synovial leukocytosis, and neutrophil percentage which can also rise in acute crystal arthropathy. Because of this, the pooled OR for PJI in our meta-analysis might partly capture misdiagnosed flares rather than true infection; it therefore warrants cautious interpretation considering the potential misclassification bias.

No significant difference was observed in transfusion requirements or in-hospital mortality between patients with and without crystallopathies in our analysis. The pooled OR for transfusion was 1.11 (95% CI: 0.90–1.37; *P* = 0.31), and for 0.69 for mortality (95% CI: 0.46–1.02; *P* = 0.054). This might show that despite the chronic inflammatory state, possibly leading to chronic disease anemia, these factors do not significantly influence the risk of transfusion need due to surgical blood loss or early postoperative mortality. The odds of these two outcomes not being significantly higher despite the existence of possible and plausible physiological mechanisms by which crystallopathies might have led to a higher risk in these two adverse events might also be due to standardized and effective perioperative care protocols, which prevent these adverse outcomes from happening. There was also no observed difference in arthropathy type and arthroplasty region in terms of the odds of transfusion need or in-hospital mortality.

A significantly increased risk of thromboembolic events was observed in patients with crystallopathies (OR: 1.19; 95% CI: 1.06–1.33; *P* = 0.0038); however, the risk of MI was not significantly different (OR: 1.10; 95% CI: 0.79–1.52; *P* = 0.57). The difference between these two conditions was, however, not significant (*P* = 0.13). Different sites of arthroplasty (TKA and THA) also did not differ significantly in this instance (*P* = 0.34). Systemic inflammation, which we previously established is present in crystal arthropathy patients, is known to dysregulate the coagulation mechanisms; this happens by activating the coagulation cascade and platelet aggregation, causing endothelial dysfunction, which can collectively elevate thromboembolism risk together^[27]^. Hyperuricemia, the culprit behind gout, has also been associated with vascular dysfunction, which can increase the risk of thromboembolism^[28]^. NSAIDs, colchicine, and corticosteroids are the generally accepted drugs for management of gout in arthroplasty patients. Allopurinol or febuxostat is continued for urate lowering, while colchicine or NSAIDs prevent flares if not contraindicated. In severe or refractory cases, corticosteroids are used short-term, but risks of infection must be considered when choosing the right drug regimen.

The odds of MI being non-significant might be interpreted as, first, effective perioperative, intraoperative, and postoperative care protocols, preventing serious cardiac events, and second, the pathophysiologic mechanisms of crystal arthropathies not seriously affecting cardiovascular outcomes.

We found statistically significant increase in non-home discharge among patients with crystallopathies (OR: 1.20; 95% CI: 1.02–1.40; *P* = 0.0276), while no significant difference was observed in 90-day emergency department (ED) visits (OR: 0.96; 95% CI: 0.64–1.45; *P* = 0.85). This relatively small increase in non-home discharge can be caused by patients being referred to other facilities to continue their treatment for their arthropathies or some comorbidities that are commonly seen in these patients.

Our analysis did not show statistically significant differences in LOS or hospital charges between patients with and without crystallopathy. The SMD in LOS was 0.03 (95% CI: − 0.0045 to 0.0679; *P* = 0.08), and for hospital charges, 0.006(95% CI:—0.0086 to 0.0209; *P* = 0.41. The wide confidence intervals, despite the higher mean, might be a sign of variability across institutional practices and patient populations because patients with gout or CPPD can be considered to require longer inpatient monitoring due to risks of flares, impaired wound healing, or management of comorbidities; in addition, it is expected that these patients require an increased amount of lab tests, imaging studies, consultations, and rehabilitations due to the possible signs and symptoms caused by the hyper inflammatory state and the limited activity caused by gout or CPPD flares which can increase total hospital costs.

There are other possible factors that might affect the extent to which crystalopathies might alter postoperative outcomes: severity of disease, frequency of flares, whether the patient is consuming any medication for their disease and if, the drugs patient is using, other comorbidities, serum levels of urate in gout, and both radiologic and functional burden and impairment of disease for the patient^[9,26,29,30]^. Current literature has not focused on these possible covariates, which calls for further studies and a personalized care plan for patients with this condition. There was a substantial amount of heterogeneity in our results; this can be caused by variations in study designs, diagnostic criteria of crystallopathies, outcome and exposure definitions, patient comorbidities, surgical procedures, and follow-up durations and protocols. Differences in gout and CPPD profiles, perioperative management, and healthcare settings and practice guidelines also add to the variability in effect estimates.

Our study is one of the most comprehensive meta-analyses examining postoperative outcomes in patients with crystallopathies undergoing total joint arthroplasty. By pooling data across a relatively large patient population, we assessed the impact of both gout and CPPD on a wide range of surgical complications. Subgroup analyses by crystal type and procedure (THA and TKA) allowed for a better understanding of possible contributing risk factors.

However, our study has several limitations. First, the included studies were mostly retrospective and heterogeneous, with variation in outcome and exposure definitions, data sources, and follow-up durations. Second, most relied on administrative or insurance claims databases, which are prone to errors and lack detailed clinical data such as flare frequency, serum urate levels, medication use, or other possible contributing factors mentioned before; this requires future prospective studies with more granular data on the subject. Third, the possible confounding bias caused by comorbidities and unmeasured variables may influence outcomes despite subgroup analyses. Significant publication bias was observed in ED visits variable, which limits the reliability of our results.

Lastly, significant heterogeneity was present in some pooled estimates.

## Conclusion

To conclude, our meta-analysis showed that patients with crystallopathies are at increased risk of certain postoperative complications after arthroplasty, including wound complications, periprosthetic joint infection, thromboembolic events, and non-home discharge. However, wound complications, thromboembolic events, and non-home discharge lost significance in sensitivity analysis. These findings underscore the importance of preoperative risk assessment and personalized peri-, intra-, and postoperative management in this patient population. Further research is needed to speculate the role of disease severity, activity, comorbidities, and flare prevention interventions in optimizing surgical strategies for patients with crystallopathies.

## Data Availability

None.
